# *Ginkgo biloba* L. Leaf Extract Protects HepG2 Cells Against Paraquat-Induced Oxidative DNA Damage

**DOI:** 10.3390/plants8120556

**Published:** 2019-11-29

**Authors:** Amélia M. Silva, Sandra C. Silva, Jorge P. Soares, Carlos Martins-Gomes, João Paulo Teixeira, Fernanda Leal, Isabel Gaivão

**Affiliations:** 1 Department of Biology and Environment, University of Trás-os-Montes e Alto Douro (ECVA, UTAD), Quinta de Prados, 5001-801 Vila Real, Portugal; scmsp_88@hotmail.com (S.C.S.); camgomes@utad.pt (C.M.-G.); 2 Centre for the Research and Technology of Agro-Environmental and Biological Sciences, (CITAB-UTAD), Quinta de Prados, 5001-801 Vila-Real, Portugal; 3 Department of Genetic and Biotechnology, (ECVA, UTAD), Quinta de Prados, 5001-801 Vila-Real, Portugal; fleal@utad.pt; 4 Research Center in Sports, Health Sciences and Human Development, ECVA, UTAD, Quinta de Prados, 5001-801 Vila Real, Portugal; 5 National Health Institute Dr. Ricardo Jorge (INSA), Rua Alexandre Herculano 321, 4000-055 Porto, Portugal; jpft12@gmail.com; 6 EPIUnit—Instituto de Saúde Pública da Universidade do Porto, Rua das Taipas, 135, 4050-091 Porto, Portugal; 7 BioISI—Biosystems & Integrative Sciences Institute, University of Trás-os-Montes and Alto Douro (BioISI-UTAD), Quinta de Prados, 5000-801 Vila Real, Portugal; 8 The Veterinary and Animal Research Centre, (CECAV-UTAD), 5000-801 Vila Real, Portugal

**Keywords:** *Ginkgo biloba* L., aqueous leaf extract, paraquat, comet assay, genotoxicity, HepG2 cells

## Abstract

*Ginkgo biloba* L. leaf extracts and herbal infusions are used worldwide due to the health benefits that are attributed to its use, including anti-neoplastic, anti-aging, neuro-protection, antioxidant and others. The aim of this study was to evaluate the effect of an aqueous *Ginkgo biloba* extract on HepG2 cell viability, genotoxicity and DNA protection against paraquat-induced oxidative damage. Exposure to paraquat (PQ), over 24 h incubation at 1.0 and 1.5 µM, did not significantly reduce cell viability but induced concentration and time-dependent oxidative DNA damage. *Ginkgo biloba* leaf extract produced dose-dependent cytotoxicity (IC_50_ = 540.8 ± 40.5 µg/mL at 24 h exposure), and short incubations (1 h) produced basal and oxidative DNA damage (>750 and 1500 µg/mL, respectively). However, lower concentrations (e.g., 75 µg/mL) of *Ginkgo biloba* leaf extract were not cytotoxic and reduced basal DNA damage, indicating a protective effect at incubations up to 4 h. On the other hand, longer incubations (24 h) induced oxidative DNA damage. Co-incubation of HepG2 cells for 4 h, with *G. biloba* leaf extract (75 µg/mL) and PQ (1.0 or 1.5 µM) significantly reduced PQ-induced oxidative DNA damage. In conclusion, the consumption of *Ginkgo biloba* leaf extract for long periods at high doses/concentrations is potentially toxic; however, low doses protect the cells against basal oxidative damage and against environmentally derived toxicants that induce oxidative DNA damage.

## 1. Introduction

Plants and herbs have been used since ancient times and today many people still rely on medicinal plants for their primary health care, others use them as a natural source of bioactive molecules. *Ginkgo biloba* L., the oldest living tree, has a long history of use in traditional Chinese medicine; nowadays the aerial parts (used as an infusion) and the standardized leaf extract (EGb761)- of *G. biloba* are among the most widely sold phytomedicines or dietary supplements in Europe and the USA [1,2]. Several benefits have been attributed to its use, including the treatment of early-stage symptoms of Alzheimer disease, cardiovascular and Raynaud’s disease, fatigue, anxiety, and depression [1,2,3,4,5], as well as its anticancer [6], anti-aging [7] and neuroprotective [5] properties. Underlying these effects are an improvement in cerebral blood flow [8], antagonism of the platelet-aggregation factor (PAF) receptor [2,5], inhibition of β-amyloid aggregation [1,5], and anti-inflammatory [9] and antioxidant activity [5], among others. Its pharmacological properties are attributed to its chemical composition, namely, to terpenoids (e.g., ginkgolides A, B, C, J), flavonoids (e.g., quercetin, rutin), biflavonoids (e.g., ginkgetin), organic acids (e.g., ginkgolic acid), and others [7,10,11,12].

The bioactivity of natural compounds such as antioxidant, anticancer, and antiaging activity, has gained importance in the phytopharmaceutical industry due to the growing interest in natural compounds for application in preventive medicine. Data on the antioxidant activity of *Ginkgo biloba* leaf extract are inconsistent; some authors highlight its ability as an antioxidant with the ability to scavenge hydrogen peroxide [13,14], as a protector against membrane lipid peroxidation [15], and its ability to increase reduced glutathione (GSH) levels [5]; others have suggested that the *G. biloba* extract is pro-oxidant [16,17] or even cytotoxic [3,18,19], which can probably be attributed to some compounds present in the whole leaf extract that may act per se or in combination in a synergistic way. These differences may arise from the methodology used, i.e., indirect methods, such as the assessment of antioxidant enzymes, studying single compounds present in the extract, or directly measuring the antioxidant activity of the extract or its fractions.

Concerning DNA repair, Marques and collaborators [14] showed an improvement in DNA repair of *Saccharomyces cerevisiae* following an oxidative shock, when pre-exposing or simultaneously exposing *S. cerevisiae* with *G. biloba* water extract in the mg/mL range of concentration. In an in vivo study, a commercially available *G. biloba* extract (EGb) was shown to reduce comet tail intensity of rat hepatocytes in a hepatocellular carcinoma model using *N*-nitrosodiethylamine as a tumor inductor [20].

Paraquat (1,1’-dimethyl-4,4’-bipyridinium dichloride) is a non-selective herbicide, which is still used even though its use is restricted, or even forbidden in some countries, and exposure to it may increase the risk of cancer [21] as it induces DNA damage in a dose-dependent fashion [22]. In this work, we aimed to evaluate the effect of *G. biloba* leaf extract in DNA and its potential protective effect against paraquat-induced DNA oxidative damage in HepG2 cells. We used HepG2 cells that retain the enzymatic activity of normal human hepatocytes and mimic in vivo responses [23], and the highly sensitive comet assay technique to assess strand breaks and oxidative damage by including the extra step of digesting the nucleoids with formamidopyrimidine-DNA-glycosylase (FPG) enzyme. This lesion-specific endonuclease converts oxidized purines, including the major purine oxidation product 8-oxoguanine as well as other altered purines (ring-opened purines or formamidopyrimidines) into DNA single strand breaks [24].

## 2. Results and Discussion

### 2.1. Cell Viability upon PQ or G. biloba Extract Exposure

**Figure 1:**
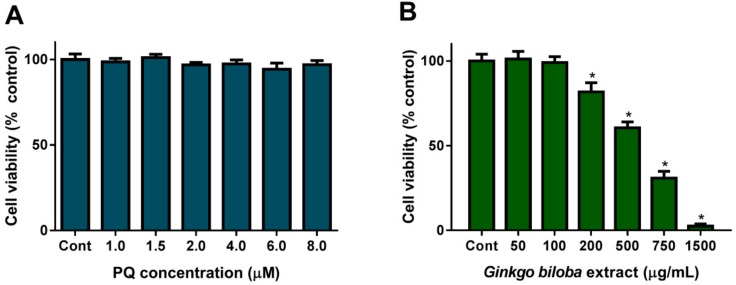
HepG2 cells’ viability upon 24 h exposure to paraquat (PQ) (**A**) and to *Ginkgo biloba* leaf extract (**B**). Cell viability was assessed using Alamar Blue assay (see Methods), data are expressed as % of control (Cont.; non-treated cells) and are shown as mean ± S.D (*n* = 4). Values that are statistically different (*P*-value < 0.05) from control are indicated by an *.

In this work, we aimed to use PQ as an oxidative stress inducer that causes significant DNA damage without producing a reduction in cell viability. Exposure of HepG2 cells to PQ (1.0–8.0 µM) for 24 h did not alter cell viability, as measured by the cell viability indicator Alamar Blue (Figure 1A). These results are in agreement with others obtained in other cell models, for example, in human neural progenitor cells (hNPCs), a slight reduction on cell viability (~95% cell viability) was observed after 24 h exposure to 10 µM PQ [25], but 90% cell viability was observed upon exposure of rat mesencephalic dopaminergic neuronal N27 cells [26] or HepG2 [27] to 100 µM PQ. Apart from cell viability, some authors reported that PQ (at 50 µM) greatly increased the production of reactive oxygen species (ROS) [28], or at concentrations as low as 1 µM, PQ significantly increased DNA strand breaks on freshly isolated alveolar macrophages [29]. Thus, in this study we chose concentrations up to 5 µM to ensure cell viability of about 100% (Figure 1A) and to ensure that ROS production and DNA damage was induced.

Concerning the effect of *G. biloba* extract on cell viability, cells were exposed for 24 h (Figure 1B) to concentrations up to 1500 µg/mL (please see Section 4.1 and Section 4.2 for experimental details). As observed, cell viability was reduced with the increasing concentration of the extract. In this experiment, we observed an IC_50_ of 540.8 ± 40.5 µg/mL after exposure for 24 h, corroborating previous works that reported the cell toxicity effect of *G. biloba* extract on HepG2 cells [30]. Figure 1B shows that concentrations up to 100 µg/mL were totally safe. Thus, a concentration lower than 100 µg/mL was chosen for the next experiments.

### 2.2. Paraquat Induces DNA Damage in HepG2 Cells

**Figure 2:**
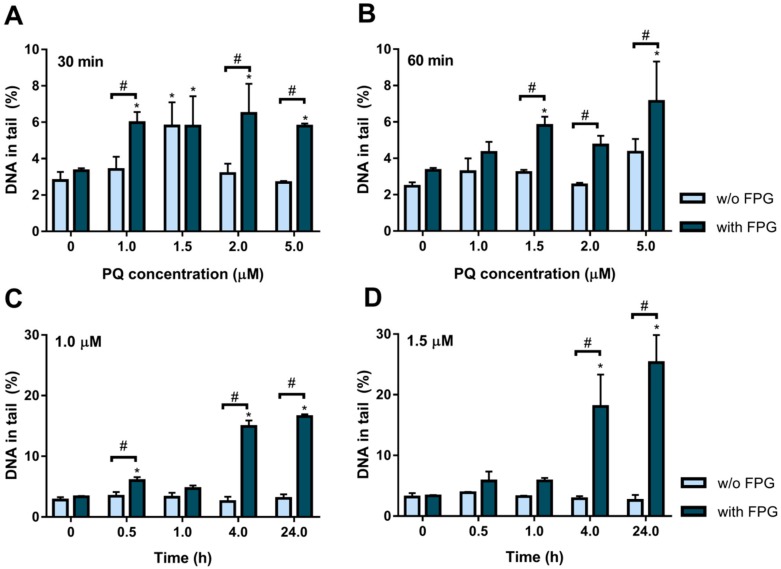
Optimization of PQ-induced DNA damage conditions, measured as % of DNA in the tail. First, HepG2 cells were incubated for 30 min (**A**) or 60 min (**B**) with different concentrations (0 (control), 1, 1.5, 2 and 5 µM) of PQ and then were submitted to comet assay in the absence (w/o) and in the presence (with) of formamidopyrimidine-DNA-glycosylase (FPG). Effect of incubation time for 1 µM PQ (**C**) and 1.5 µM PQ (**D**), DNA strand breaks in the absence of enzyme (light blue bars, w/o FPG), and in the presence of FPG (dark blue bars, with FPG), are shown as mean values of three different independent experiments ± SEM. The asterisk (*) means significant differences compared to the respective control. The # means significant differences between treatment in the presence and absence of FPG. *P*-value < 0.05.

We aimed to study the effect of PQ as an inducer of DNA damage in HepG2 cells, and without compromising cell viability, we evaluated the % of DNA in the tail of cells that were first exposed to PQ and then subjected to comet assay (please see Methods). Figure 2 shows the results obtained for PQ-induced DNA damage in HepG2 cells after 30 and 60 min (Figure 2A,B, respectively) exposure to different concentrations (1.0, 1.5, 2.0 and 5.0 μM PQ). As observed, 30 min (Figure 2A) incubation with 1.5 μM PQ increased DNA damage, strand breaks (light blue bar; 5.79 ± 1.30% of DNA in the tail), and oxidative damage (dark blue bar; 5.78 ± 1.63%). Similar oxidative damage was also observed at 1.0 μM (dark blue bars; 5.98 ± 0.58%). Higher concentrations of PQ did not result in a significantly higher amount of DNA damage (Figure 2A). Longer exposure to PQ, for 1 h incubation (Figure 2B), showed an increase in oxidative damage (dark blue bars) for 1.5 and 5.0 μM PQ, compared to the respective control. However, strand breaks (light blue bars) were not different from the control, which may be a result of internal cell repair mechanisms.

In a parallel study, two PQ concentrations, 1.0 μM (Figure 2C) and 1.5 μM (Figure 2D) were assayed on DNA damage along time, namely, at 0.5, 1, 4 and 24 h incubation. Over time, 1.0 µM PQ did not induce significant strand damage, in comparison to control (*P* > 0.05). But, although the level of oxidized purines remained similar to control (*P* > 0.05) at 1 h treatment, it peaked at 4 h of treatment (dark blue bar; 14.92 ± 0.96% of DNA in tail), with this value being 4.5-fold higher on average, than control (*P* < 0.05). The % DNA in the tail at 24 h (dark blue bar; 16.53 ± 0.37) is slightly higher than at 4 h, but the differences are statistically significant (*P* < 0.05; Figure 2C). For 1.5 µM PQ (Figure 2D), the results show a similar pattern, that is, the maximum oxidative damage at 24 h incubation (dark blue bar; 25.28 ± 4.60%) is ~7.6-fold higher than control.

Paraquat is an oxidizing molecule that has been shown to induce DNA damage in some studies (e.g., mice hepatocytes [31] alveolar macrophages [29], Caco-2 cells [32]). The hypothesized mechanism involves PQ reduction by NADPH-oxidase, decreasing the levels of NADPH, which is oxidized to NADP. Reduced PQ is regenerated into oxidizing-PQ with the production of a superoxide radical (O_2_^•^^−^), which may be converted into hydrogen peroxide (H_2_O_2_) by superoxide dismutase (SOD) and then eliminated by catalase (CAT). The activity of these enzymes determines the production of oxygen reactive species (ROS) that contribute to DNA damage [31,33,34,35]. In the present study, PQ concentration and exposure time were optimized for HepG2 cells. Results show that damage is concentration and time-dependent, which could be attributed to PQ inducing lipid peroxidation and peroxyl radicals, which then contribute to DNA damage, as described previously [33,36,37,38]. Also, PQ can increase oxidative stress and mitochondrial damage [38]. The values of DNA damage for 1 µM PQ were identical from 4 to 24 h, which may suggest that the produced radicals did not injure the repair enzymes that attempt to restore the initial sequence of DNA. 

### 2.3. Effect of Ginkgo biloba Extract on DNA Damage

**Figure 3:**
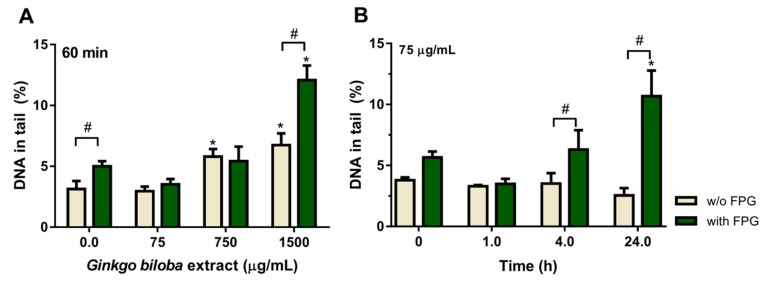
Effect of *Ginkgo biloba* leaf extract in DNA damage measured by comet assay as a percentage of DNA in the tail. (**A**) Effect of different concentrations, 0 (control), 75, 750 and 1500 µg/mL of *G. biloba* extract for 1 h incubation. (**B**) Effect of 75 µg/mL *G. biloba* extract, for 1, 4 and 24 h of exposure. DNA strand breaks in the absence of enzyme (light grey bars, w/o FPG), and in the presence of FPG (green bars, with FPG) are shown by mean values ± S.E.M. The asterisk (*) means significant differences compared to the respective control. The # means significant differences between treatment in the presence and absence of FPG. *P*-value < 0.05.

Concerning the effect of *Ginkgo biloba* extract by itself, on DNA damage, was evaluated using the following concentrations: 75, 750 and 1500 µg/mL and exposure periods: 5 min and 30 min, and 1 h, 4 h and 24 h. Figure 3A presents the effect of 1 h exposure to various concentrations of *G. biloba* extract, and shows the dose-dependent DNA damage for higher concentrations, which is more evident when measured as oxidative damage (with FPG). Concentrations of 750 and 1500 µg/mL significantly increased strand breaks (light grey bars) compared to control, and oxidized purines (green bars) attain a higher percentage for 1500 µg/mL of extract (~2.4-fold higher than control, *P* < 0.05). However, at 75 µg/mL the extract did not induce DNA damage for 1 h exposure (Figure 3A), it even showed a reduction in oxidative damage, thus this concentration was used to study the damage over time (Figure 3B). As can be seen in Figure 3B, 75 µg/mL *G. biloba* extract over time did not significantly affect DNA damage, which was evaluated by strand breaks (*P* > 0.05), and its effect on oxidative damage (with FPG) only shows a significant increase in oxidized purines at 24 h incubation (~2-fold higher than control). These results are in line with the results of cell viability (Figure 1A), and corroborate the results reported by Grollino et al., in which no DNA damage was detected by conventional comet assay (strand breaks analysis) in HepG2 cells exposed to *Ginkgo biloba* extract (IDN5933, Ginkgoselect®Plus) at concentrations up to 2 mg/mL. These authors, pointed out that cell toxicity is a result of oxidative stress [39], and here we report that *Ginkgo biloba* aqueous extract at higher concentrations induces oxidative DNA damage (Figure 3B). Thus, high concentrations of *Ginkgo biloba* aqueous extract induced DNA damage, suggesting the direct and nonspecific harmful effects of *G. biloba* leaf extracts in DNA when used at high concentrations. Babich and co-workers [17] reported that polyphenols can injure DNA depending on their concentration. *G. biloba* compounds that may account for this genotoxicity include ginkgetin [40], quercetin [41] and ginkgolic acids [18,19,42], which contribute as individual compounds depending on their concentration in the extract [43].

Hecker and co-workers [19] reported the release of lactate dehydrogenase (LDH) from human keratinocyte (HaCaT) and in rhesus monkey kidney tubular epithelial cell line (LLC-MK2) incubated with *Ginkgo* extract (EGb 761), which is indicative of loss of membrane integrity. Other studies have suggested that the pro-oxidant activity of *G. biloba* extract causes cell toxicity [16,39,43]. Future studies should explore the involvement of oxidative stress enzymatic system to long-term treatment with *G. biloba* extract.

From the above experiment, we chose to use 75 µg/mL *G. biloba* extract and 4 h incubation, as this concentration and period of incubation do not cause cell toxicity and DNA damage (basal and oxidative).

### 2.4. Ginkgo biloba Extract Protects HepG2 Cells Against Paraquat-Induced DNA Damage

**Figure 4:**
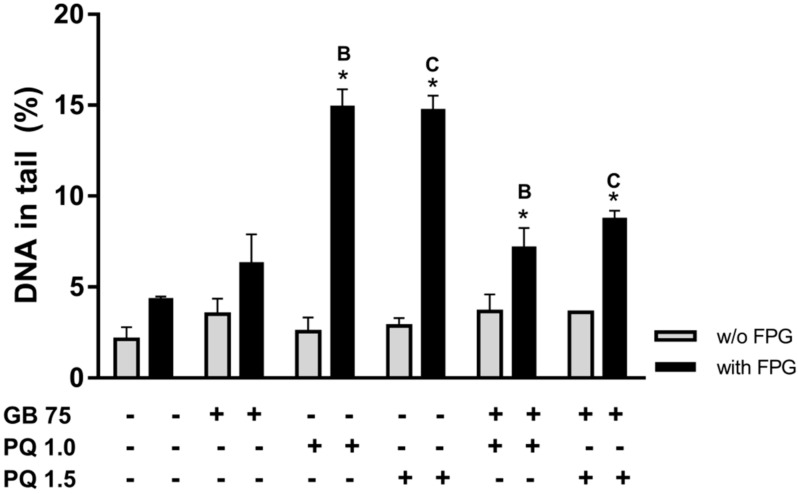
Effect of *Ginkgo biloba* extract against paraquat-induced DNA damage. Percentage of DNA in the tail of comets obtained from cells incubated with *G. biloba* extract at 75 µg/mL (GB 75), with 1.0 or 1.5 µM paraquat (PQ 1.0 or PQ 1.5, respectively), and for simultaneous exposures (75 µg/mL *G. biloba* plus PQ 1.0 or 1.5 µM) for 4 h, as denoted (+ and −, means the presence or absence of). DNA strand breaks in the absence of enzyme (grey bars) and in the presence of FPG (black bars) are shown by mean values ± S.E.M. The asterisk (*) means significant differences compared with the respective control (non-exposed cells). The same letter means significant differences between treatments. *P*-value < 0.05.

From the above experiment, we chose to use 75 µg/mL *G. biloba* extract and 4 h incubation, because this concentration and period of incubation do not cause cell toxicity or DNA damage with significant differences. HepG2 cells were concomitantly exposed to 75 µg/mL *G. biloba* and to 1.0 or 1.5 µM PQ (Figure 4) for 4 h. As can be seen in Figure 4, exposure to 75 µg/mL *G. biloba* aqueous extract for 4 h shows no statistically significant DNA damage. However, by itself, 1.0 or 1.5 µM of PQ significantly increases DNA oxidative damage (Figure 4), as observed in Figure 2. Co-exposing cells, for 4 h to 75 µg/mL *G. biloba* extract plus 1.0 or 1.5 µM PQ, results in a significant reduction in PQ-induced oxidative purines (*P* < 0.05). 

Our findings corroborate the results of other experimental models, such as in the case of simultaneous incubation of *Saccharomyces cerevisiae* with *G. biloba* water extract plus hydrogen peroxide, where *G. biloba* showed a DNA protective effect [14]. 

The pro-oxidant potential of *G. biloba* extract at high concentrations, which may induce mutations in the DNA sequence that will join to naturally occurring mutations, is of interest due to concerns about the potential health risks for the general population who are exposed to diet supplements. Over time, cell antioxidant defenses weaken or become inadequate, increasing the risk of developing genetically-based diseases, such as cancer. A study in Swiss albino mice showed that long-term treatment with *G. biloba* extract can cause aneuploids, decreased fertility and hampers the pre-implantation of embryos due to embriotoxic and neurotoxic effects. This also warrants its careful use as a remedy for male impotence and/or erectile dysfunction [3]. Some recent studies highlight some negative results from relatively large clinical trials on the frequent consumption of *G. biloba* for long periods of time [43]. We have also observed that high doses of *G. biloba* extract are cytotoxic (Figure 1A) and cause oxidative DNA damage (Figure 3). 

Transposing this study to the consumer point of view, a tea-bag contains about 1.5 g of dry material which serves to make one cup of infusion (~250 mL). Taking into consideration that most of the compounds in the infusion will suffer pH degradation (in the stomach and intestine) and/or oxidation, and that absorption will not be complete as some compounds will not be absorbed because of the presence of fibers in the gut, we may speculate that the amount of extract reaching the whole body is in the antioxidant range. However, caution must be taken if several *G. biloba* infusions are consumed per day on a continuous basis. Although, the standardized *G. biloba* supplements have a known composition and are composed of selected compounds that are beneficial to the human organism, consumers should also pay attention to the manufacturer’s recommendations for dosage and not overconsume these supplements. 

## 3. Conclusions

Paraquat may be used experimentally as oxidative stress inducer. The aqueous extract of *Ginkgo biloba* leaf at low concentrations (up to 200 µg/mL), did not produce cell toxicity or induce DNA damage for short exposures, but instead resulted in a reduction in oxidative DNA damage, indicating an antioxidant effect. However, at higher concentrations, cell toxicity and oxidative DNA damage were observed in a concentration and time-dependent manner. Most importantly, at low concentrations, i.e., as found in the regular usage of a tea-bag per day, a protective effect against PQ-induced DNA damage was observed. Given these results, an antioxidant effect for low concentrations and oxidant properties of *Ginkgo biloba* extract at high concentrations were observed, thus care should be taken when using frequent infusions or extracts directly from leaves for long periods and at high dosages. The moderate consumption of *G. biloba* infusions might contribute flavonoids and other phenolic compounds that act as free radical scavengers to reduce cell oxidative stress. However, high doses, in addition to providing beneficial compounds, also appear to contribute compounds that generate cellular stress, which can be translated into DNA damage. PQ was used as a model for oxidative DNA damage, further studies could examine the protective effect of *Ginkgo biloba* extract (at low doses) against other environmental stressors, such as food containing contaminants (e.g., pesticides, heavy metals).

## 4. Materials and Methods 

### 4.1. Preparation of Ginkgo biloba Aqueous Extract

*Ginkgo biloba* leaves were collected at Vila Real, Portugal from a tree sample in the University Botanical Garden (voucher ID: HVR4565, UTAD, Quinta de Prados, Vila Real; WGS84 103 (41.2884381; 7.739039). Aqueous extraction was performed as described by Ding and co-workers in [44]. Briefly, the leaves were washed with distilled water, air-dried at room temperature in the shade and then powdered. Then, 30 mL of boiling distilled water was added to 3.6 g of plant material and this mixture was heated in a water bath at 100 °C for 5 min and then centrifuged at 2000 ×*g* for 15 min. The supernatant was collected, filtrated, the pH adjusted to 6.5 and it was then frozen at −20 °C before its lyophilization. After lyophilization, a yield of 15.3% ((dried extract weight/ dried plant material) × 100%) was obtained.

### 4.2. Cells Culture and Experimental Design

#### 4.2.1. Cell Culture and Maintenance 

The human hepatoma cell line (HepG2; ATCC, Rockville, MD) was maintained in culture in DMEM (Dulbecco’s modified Eagle’s medium, Gibco, Life Technologies) with 25 mM glucose, supplemented with 10% fetal bovine serum (FBS; Gibco, Life Technologies), 2 mM L-glutamine (Gibco, Life Technologies) and antibiotics (100 U/mL penicillin and 100 µg/mL of streptomycin, Gibco, Life Technologies) in an atmosphere of 5% CO_2_ in air at 37 °C, as described previously [45,46].

#### 4.2.2. Cell Viability/Cytotoxicity Assay 

HepG2 cells were handled as described in [45,46]. Briefly, HepG2 cells were seeded in 96-well plates, at 5 × 10^4^ cells/mL (100 µL/well) for 24 h. After seeding, the culture media was replaced by FBS-free culture media supplemented with PQ (1–8 µM) or with *G. biloba* extract at the desired concentration (from 50 to 1500 µg/mL). To prepare these solutions, a 20 mg/mL stock solution of *G. biloba* extract was prepared in FBS-free culture medium, and from this, subsequent dilutions were made in FBS-free culture medium. Cell viability was evaluated with Alamar blue assay (Alfagene, Invitrogen, Portugal). Thus, 24 h after exposure of the cells, FBS-free culture media supplemented with 10% (v/v) of Alamar blue was added (100 µL to each well), and absorbance at 570 nm (reduced form; resorufin) and 620 nm (oxidized form; resazurin) was read ~4 h later. Data were analyzed by calculating the percentage of Alamar blue reduction (according to the manufacturer’s recommendation) and expressed as a percentage of control (untreated cells), as reported in [47].

#### 4.2.3. Cell Treatment and Experimental Conditions for Comet Assay 

HepG2 cells were seeded onto 12-well culture plates at a density of 1 × 10^5^ cells/mL (1 mL/well). After seeding for 24 h, the culture medium was discarded and replaced by *Ginkgo biloba* extract and/or paraquat (at desired concentrations, please see the Results section for details) diluted in FBS-free culture medium. Negative controls were made throughout using FBS-free culture medium. 

Treatments were performed in three distinct conditions: (i) For optimization of the binomial concentration-time to maximize PQ-induced oxidative stress, cells were incubated with PQ at final concentrations of 1.0, 1.5, 2.0 and 5.0 µM for 30 min or 1 h, and for lower PQ concentrations (1.0 and 1.5 µM), incubations of 0.5, 1, 4 and 24 h were used. (ii) To study the effect of *G. biloba* extract on DNA damage, cells were exposed to 75, 750 and 1500 µg/mL of *G. biloba* extract (stock solution diluted in FBS-free culture media) for 1 h, and for 75 µg/mL of *G. biloba* extract, comet assay was performed at 1, 4 and 24 h after cell exposure. (iii) Finally, a set of cells were submitted to *G. biloba* extract (75 µg/mL) incubation for 4 h and simultaneously incubated (co-incubation) with 1.0 or 1.5 µM PQ.

### 4.3. Alkaline Comet Assay

Comet assay was performed as described previously [29,34,48,49]. Briefly, after treatment, cells were washed with PBS, treated with trypsin, subjected to centrifugation and washed twice with PBS. Cells were dispersed in 150 μL of 1% (*w*/*v*) low-melting agarose (Gibco, Life Technologies) in PBS, heated at 37 °C, and embedded in gels, which were placed onto glass microscope slides pre-coated with 1% (*w*/*v*) normal melting point agarose (Sigma Chemicals) in water. Each gel (70 µL) contained approximately 2 × 10^4^ cells [48].

#### 4.3.1. Measurement of Strand Breaks

The two gels, placed on each side of a microscope slide, served as duplicates. Slides with cells were immersed into cold lysis solution (2.5 M NaCl, 0.1 M EDTA, 0.01 M Tris, pH 10 adjusted with NaOH, and 1% Triton X-100, which was added immediately before use) for 1 h at 4 °C. Then, slides were placed in electrophoresis solution (0.3 M NaOH, 1 mM EDTA) for 40 min at 4 °C in the dark (alkaline treatment). Subsequently, gels underwent electrophoresis at 25 V and 300 mA, for 30 min at 4 °C in the dark. After electrophoresis, gels were washed twice with cold (4 °C) distilled water. Strand breaks allow DNA to extend from the nucleoids towards the anode, forming a “comet tail”.

#### 4.3.2. Measurement of Oxidative DNA Damage

FPG (a lesion specific enzyme) was used to measure oxidized purines, including 8-oxoguanine. After lysis the slides were washed three times with enzyme reaction buffer (0.1 M KCl, 0.5 mM EDTA, 40 mM HEPES, 0.2% (*w*/*v*) bovine serum albumin, pH adjusted to 8 with KOH) at 4 °C. After washing, 50 µL of FPG was dropped onto each gel, covered with glass and incubated at 37 °C for 30 min in a humid chamber. Alkaline treatment and electrophoresis then followed as described above. The additional breaks formed at the oxidized purines lead to an increase in the amount of DNA in the tail. 

#### 4.3.3. Comet Evaluation

Comets were visualized by fluorescence microscopy (Nikon Eclipse E400) after staining with ethidium bromide (Sigma Chemical), and computerized image analysis was applied using Comet Assay IV software. The percentage of DNA in the tail (mean value from 50 comets per gel) was taken as a measure of DNA damage.

### 4.4. Data and Statistical Analysis

Data from different experiments are presented as mean ± standard deviation (S.D.) or as mean ± S.E.M. (as mentioned). The value of IC_50_ (concentration that inhibits 50% of cell viability/proliferation or half maximal inhibitory concentration ) was calculated as reported by Silva et al [50]. Data analysis was performed using the software Statistical Program for Social Sciences (SPSS), version 20.0, as described in [49]. Statistical significance of differences between mean values was assessed by the ANOVA test (confidence level 95%; *P*-value < 0.05). The Tukey test was applied for multiple comparisons.
